# Neutralizing Antibodies Induced by Gene-Based Hydrodynamic Injection Have a Therapeutic Effect in Lethal Influenza Infection

**DOI:** 10.3389/fimmu.2018.00047

**Published:** 2018-01-24

**Authors:** Tatsuya Yamazaki, Maria Nagashima, Daisuke Ninomiya, Akira Ainai, Akira Fujimoto, Isao Ichimonji, Hidekazu Takagi, Naoko Morita, Kenta Murotani, Hideki Hasegawa, Joe Chiba, Sachiko Akashi-Takamura

**Affiliations:** ^1^Department of Microbiology and Immunology, School of Medicine, Aichi Medical University, Nagakute, Japan; ^2^Department of Biological Science and Technology, Tokyo University of Science, Katsushika, Japan; ^3^Department of Pathology, National Institute of Infectious Diseases, Shinjuku, Japan; ^4^Division of Biostatistics, Clinical Research Center, School of Medicine, Aichi Medical University, Nagakute, Japan

**Keywords:** passive immunotherapy, neutralizing antibody, gene therapy, hydrodynamics procedure, influenza, hemagglutinin

## Abstract

The influenza virus causes annual epidemics and occasional pandemics and is thus a major public health problem. Development of vaccines and antiviral drugs is essential for controlling influenza virus infection. We previously demonstrated the use of vectored immune-prophylaxis against influenza virus infection. We generated a plasmid encoding neutralizing IgG monoclonal antibodies (mAbs) against A/PR/8/34 influenza virus (IAV) hemagglutinin (HA). We then performed electroporation of the plasmid encoding neutralizing mAbs (EP) in mice muscles and succeeded in inducing the expression of neutralizing antibodies in mouse serum. This therapy has a prophylactic effect against lethal IAV infection in mice. In this study, we established a new method of passive immunotherapy after IAV infection. We performed hydrodynamic injection of the plasmid encoding neutralizing mAbs (HD) involving rapid injection of a large volume of plasmid-DNA solution into mice *via* the tail vein. HD could induce neutralizing antibodies in the serum and in several mucosal tissues more rapidly than in EP. We also showed that a single HD completely protected the mice even after infection with a lethal dose of IAV. We also established other isotypes of anti-HA antibody (IgA, IgM, IgD, and IgE) and showed that like anti-HA IgG, anti-HA IgA was also effective at combating upper respiratory tract IAV infection. Passive immunotherapy with HD could thus provide a new therapeutic strategy targeting influenza virus infection.

## Introduction

Administration of monoclonal antibodies (mAbs) is an important therapeutic method for treating a variety of diseases such as infection, cancer, and autoimmune disease. Passive immunotherapy using mAb-based products has been therapeutically and financially successful in developed markets. Classical passive immunity provides the benefit of prevention and treatment against various infectious diseases (1). However, commercial development of recent antiviral mAbs has been delayed (2), due to complex reasons including the current availability of antimicrobial drugs, small markets, high costs, and microbial antigenic variation (3). The influenza virus causes annual epidemics and occasional pandemics and continues to be one of the major public health problems. Indeed, the annual influenza epidemics result in 3–5 million cases of severe illness and 250,000–500,000 deaths worldwide (4). Development of vaccines and antiviral drugs is therefore essential to control influenza virus infection. However, logistical problems such as antigen shift often limit the efficacy of vaccine availability. Moreover, the need to administer antiviral drugs after infection limits their utility, because the viruses can acquire resistance to them (5–8).

Kalenik et al. have reviewed influenza prevention and treatment by passive immunization (9). Many researchers have cloned the various neutralizing mAbs against influenza virus and have shown a beneficial effect (9). We have previously demonstrated a novel approach for passive immune-prophylaxis against influenza virus infection (10). We generated a plasmid encoding neutralizing IgG mAbs against the hemagglutinin (HA) protein of A/Puerto Rico/8/34 (A/PR8) influenza virus (IAV). We then performed electroporation of the plasmid encoding neutralizing mAb (EP) in mice muscles and succeeded in inducing the expression of neutralizing IgG mAbs in mouse serum. We could show that a single use of this method resulted in long-term expression and complete prophylactic efficacy before lethal influenza virus infection. This was also the first report of passive immunotherapy with a non-viral vector (10). We proposed that the passive immune-prophylaxis therapy could overcome problems of high cost, limited supply, and the danger of using viral vectors.

In this study, we established a new method of passive immune-therapy and showed the presence of protective effects even after influenza virus infection. We performed hydrodynamic injection of a plasmid encoding neutralizing mAbs (HD) involving rapid injection of a large volume of plasmid-DNA solution into mice *via* the tail vein (11, 12) and demonstrated its therapeutic efficacy after a lethal dose of IAV infection. HD could rapidly induce recombinant antibodies in the serum and some mucosal organs. Furthermore, we established other isotypes (IgA, IgM, IgD, and IgE) of anti-HA antibodies to prevent IAV infection of the upper respiratory tract because there is a great difference in the immunological function of each antibody isotype. Several studies have succeeded in preventing upper respiratory tract infection by passive intravenous injection of secretory IgA, but not IgG (13–15). Joining chain positive IgM can be transported to the mucosal lumen *via* a polymeric Ig receptor (pIgR) (16). IgE interacts with cells such as mast cells and basophils that express the high affinity IgE receptor (FcεRI) and show protective functions against parasites (17). A previous report indicated that IgD production occurred in the upper respiratory mucosa (18). It also demonstrated that IgD-stimulated basophils produced antimicrobial factors and contributed to reduction of bacterial growth (18). However, it was unclear whether passive injection of anti-HA IgM, IgD, and IgE could prevent IAV infection of the upper respiratory tract. In the current study, we show that in addition to anti-HA IgG, anti-HA IgA was also effective at combating upper respiratory tract infection, although anti-HA IgM, IgD, and IgE were ineffective. Thus, passive immunotherapy with HD could provide a new therapeutic strategy targeting influenza.

## Materials and Methods

### Mice

Female BALB/c mice (5–7 weeks old) were purchased from Sankyo Laboratories (Tokyo, Japan) or SLC (Shizuoka, Japan) and maintained in the animal facility at Tokyo University of Science and Aichi Medical University. All animal experiments were performed according to the guidelines of the Tokyo University of Science and Aichi Medical University.

### Plasmid Construction

In our previous report (10), we generated plasmids encoding the genes for the heavy chain (IgG) and the light chain (kappa) of a neutralizing anti-HA antibody (19) (Data S1 in Supplementary Material). In the current study, all constructions are based on the pCADEST1 vector, which was constructed from pCA5, a CAG promoter-driven plasmid, and pDEST12.2 (Invitrogen) (20). Mouse IgA, Joining chain, and IgE were cloned from spleen cells. IgM and IgD cDNA were cloned from the mouse B-cell line, WEHI-231. All the sequence data were described in Data S1 in Supplementary Material. Each plasmid vector encoding the anti-HA antibody was constructed by overlap PCR from pCADEST1-anti-HA IgG1 using the primers indicated in Table S1 in Supplementary Material. The plasmids, pCADEST1-anti-HA IgG1, pCADEST1-anti-HA IgA, pCADEST1-anti-HA kappa chain, and pCADEST1-Joining chain were purified by CsCl-ethidium bromide gradient centrifugation (21). pCADEST1-anti-HA IgM, pCADEST1-anti-HA IgD, and pCADEST1-anti-HA IgE were purified using NucleoBond^®^ kits (Clontech, CA, USA) according to the manufacturer’s instructions.

### ELISA

The procedure for measuring the anti-HA antibody levels was described previously (10, 22). Briefly, a 96-well plate was coated with HA protein purified from influenza virus [A/Puerto Rico/8/34 (A/PR8); H1N1] using affinity columns constructed by coupling of CNBr-activated Sepharose 4B beads (GE Healthcare UK Ltd., Buckinghamshire, England) and anti-HA antibodies. The plate was then incubated with PBS containing 25% Block Ace^®^ (Snow Brand Milk Products, Tokyo, Japan) for blocking. After washing, the plate was incubated with serially diluted mouse serum samples. Sera containing HA-specific antibodies were detected using alkaline phosphatase-conjugated goat anti-mouse IgG1 (Southern Biotech Birmingham, AL, USA) or an anti-mouse kappa chain (Southern Biotech). *p*-Nitrophenyl phosphate (Wako, Tokyo, Japan) was used as the chromogenic substrate. Absorbance was measured using AUTO READER III (Sanko-junyaku, Tokyo, Japan). Anti-HA IgG purified from a hybridoma (19) was used as the standard (10, 22). HRP-conjugated goat anti-mouse IgG (Southern Biotech), anti-mouse IgA (Thermo Fisher Scientific), anti-mouse IgM (Southern Biotech), and anti-mouse IgE (Southern Biotech) were purchased to measure anti-HA IgG, anti-HA IgA, anti-HA IgM, and anti-HA IgE in serum and nasal wash, respectively. Anti-HA IgD was measured using APC-conjugated rat anti-mouse IgD (11-26c.2a Biolegend, San Diego, CA, USA), followed by incubation with HRP-conjugated rabbit anti-rat IgG (Thermo Fisher Scientific). Finally, binding was detected using an ELISA POD Substrate TMB Kit (Nacalai Tesque, Tokyo, Japan). Absorbance was measured using Spectramax M5 (Molecular Devices, CA, USA). Secretary IgA against HA was measured using rabbit polyclonal antibodies against mouse pIgR (Sino Biological, Boston, MA, USA), followed by incubation with HRP-conjugated goat anti-rabbit IgG (BD Pharmingen, San Diego, CA, USA). We could not determine the titer of anti-HA IgD, because appropriate standards were not available.

### Competitive ELISA

For competitive ELISA shown in Figures 4C,D and 5B, we used biotin-conjugated anti-HA IgG antibodies purified from a hybridoma (19), along with HRP-conjugated streptavidin (Figure S1 in Supplementary Material).

### Western Blotting

The supernatants and standards (IgG1, IgA, IgM; Southern Biotech) (IgE; BD Pharmingen) were separated under non-reducing conditions by SDS-PAGE (6%) and transferred to a PVDF membrane (Immobilon-P) (Millipore, Billerica, MA, USA). The membrane was blocked with Blocking One reagent (Nacalai Tesque) for 30 min, followed by incubation at room temperature with a mixture of HRP-conjugated goat anti-mouse IgG (Southern Biotech), IgA, IgM, and IgE. Anti-HA IgD was measured using APC-conjugated rat anti-mouse IgD, followed by incubation with HRP-conjugated rabbit anti-rat IgG. The specific bands were visualized using the enhanced chemiluminescence substrate (GE Healthcare) on the ImageQuant LAS4000 system (GE Healthcare).

### Measurement of Antibody Expression *In Vivo*

We performed EP (10, 23–27) and HD (11, 12) using a previously described method. In EP, 30 µg of plasmid (1 µg/µl) was injected into the adductor and rectus femoris and cranial tibial muscle. Six pulses (100 V, 50 ms, polarity reversal every 3 pulses) were delivered into the injection site. All muscles were pre-treated by an injection of bovine hyaluronidase about 10 min before gene transfer to decrease the viscosity of the extracellular matrix and facilitate DNA diffusion (28). In HD (12), mice were injected in the tail vein with PBS containing plasmid (e.g., 5 μg/1.6 ml), where the DNA volume was 8–12% of the body weight. The injection was performed over less than 5 s using a 26-gauge needle. Mice in the control group were not treated (naïve group) or received gene transfer with pCADEST1-empty (vector control group). To eliminate serum from the bronchoalveolar lavage and nasal wash specimens, they were obtained directly from the respiratory tract with a 20 G indwelling needle or a bent oral sonde kindly gifted by Dr. Kiyoko Iwatsuski-Horimoto (Institute Medical of Science, University of Tokyo).

### Virus Challenge in Lethal Pneumonia

Virus challenge was carried out as described previously (10, 29). Thus, mouse adapted influenza virus (A/PR8) was grown in the allantoic cavities of 10- to 11-day-old fertile chicken eggs and stored at −80°C until used. The mice were anesthetized and intranasally challenged with 1,000 times the 50% tissue culture infective dose (TCID_50_) of A/PR8 in 20 µl PBS, which causes lethal pneumonia (40 LD_50_). Mice were subjected to gene transfer on the indicated day post-infection. Survival and weight changes were monitored for 20 days after virus challenge.

### Measurement of Virus Titer in the Bronchoalveolar Lavage

BALB/c mice that underwent gene transfer in the same manner one day after virus challenge, were prepared for estimation of virus titers in bronchoalveolar lavage specimens using a previously described method (10, 30). Three days post-infection, the bronchoalveolar lavage was obtained by washing isolated lungs with 2 ml of PBS containing 0.1% bovine serum albumin. Serial 10-fold dilutions of the specimens were inoculated onto Madin-Darby canine kidney (MDCK) cells. Three days post-infection, the MDCK cells were fixed and stained with naphthol blue black (Sigma-Aldrich, St. Louis, MO, USA). The plates were then washed and 0.1 M NaOH was added to each well. The cytopathic effect (CPE) resulting from IAV infection was evaluated by measuring the absorbance. The virus titer was calculated as TCID_50_ by the Reed–Muench method.

### Virus Challenge in the Upper Respiratory Infection Model

BALB/c mice were sublethally infected with 2 µl of PBS containing an A/PR8 virus suspension with 1,000 plaque-forming units (PFU) into each nostril (total volume of 4 µl per mouse) (29, 31). Three days post-infection, the nasal wash was obtained in 1 ml of PBS containing 0.1% bovine serum albumin. The viral titer was determined by the MDCK-plaque assay as described previously (29, 32). Briefly, MDCK cells were cultured in 6-well tissue culture plates. Upon attaining confluence, the medium was removed and serial 10-fold dilutions of the specimens were added to the cells. After 60 min, the inoculum was removed and 2 ml of agar medium containing acetylated trypsin (Sigma) was overlaid. Two days post-infection, the cells were stained with crystal violet (Nacalai Tesque), followed by counting the number of plaques to determine the viral titer in terms of PFU.

### Antibody Expression *In Vitro*

Human embryonic kidney (HEK) 293T cells were maintained in Dulbecco’s modified Eagle’s medium supplemented with 10% heat-inactivated fetal calf serum and penicillin–streptomycin–glutamine (ThermoFisher Scientific, Waltham, MA, USA). HEK293T cells were co-transfected with the indicated vector using FUGENE HD Transfection Reagent (Promega, WI, USA). After 1 week, the supernatants were obtained and used for the neutralizing assay.

### Neutralizing Assay

The neutralizing titer of the antibody-expressed supernatants was measured by a micro-neutralization assay as described previously (10). Briefly, 100 TCID_50_ of A/PR8 viruses were mixed with an equal volume of serially diluted supernatants treated with or without a receptor destroying enzyme (RDE) (Denka Seiken, Tokyo, Japan), and then incubated for 30 min at 37°C. The mixtures were then inoculated onto MDCK cells and incubated for 3 days. The CPE for IAV infection was evaluated by measuring the absorbance at 630 nm. The neutralization titer was defined as the highest dilution that demonstrated no CPE.

### Statistics

All graphs were constructed using GraphPad Prism 7 (GraphPad Software). Data were analyzed using a non-parametric Kruskal–Wallis test considering *p* < 0.05 as statistically significant. Data are shown and compared as medians and ranges of 3 or 5 mice in different groups.

## Results

### HD Induces Rapid and Stable Production of Neutralizing Antibodies

**Figure 1:**
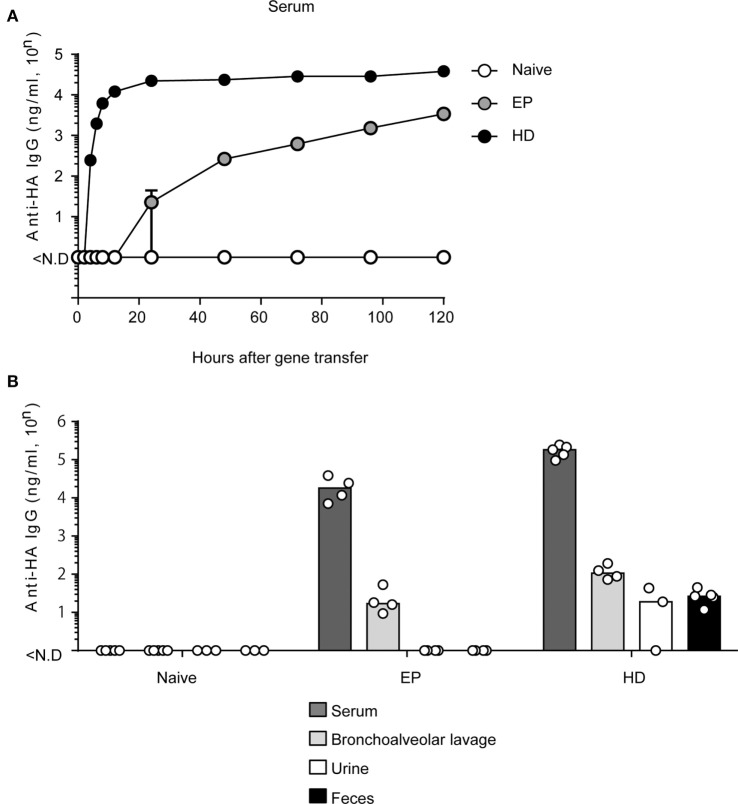
HD rapidly induces anti-HA neutralizing monoclonal antibody (mAb) in serum and in multiple mucosa. **(A)** Quantitative ELISA for neutralizing mAb in sera collected at indicated times after administration of the plasmid coding anti-HA IgG by electroporation (EP, *n* = 3) and by hydrodynamics (HD, *n* = 5). The naïve group represents no treatment (*n* = 5). Error bars represent the SEM. **(B)** Five days after HD or 20 days after EP, the serum (Naïve, *n* = 5, EP, *n* = 4, HD, *n* = 5), bronchoalveolar lavage without serum contamination (*n* = 5, *n* = 4, *n* = 4), urine (*n* = 3, *n* = 4, *n* = 3), and fecal (*n* = 3, *n* = 4, *n* = 5) specimens were obtained and subjected to quantitative ELISA. Each bar represents the median. The detection limit was over 46 ng/ml (serum) or 0.96 ng/ml (others). Each bar represents the median. ND, not detected. The indicated data about HD are representative of two independent experiments.

We previously demonstrated the potent prophylactic efficacy of EP against IAV infection (10). On the other hand, for a therapeutic effect after IAV infection, rapid and high-level production of neutralizing antibodies is indispensable. We first confirmed the expression level of anti-HA antibodies in the serum during 5 days (120 h) after EP (10) and HD. The hydrodynamics procedure involves rapid injection of a large volume of plasmid-DNA solution into the tail vein of mice (12). Several reports show that hydrodynamic injection induces peak expression at 6–8 h after gene transfer in the liver, which is a major tissue of gene expression (11, 12). As shown in Figure 1A, we could first detect anti-HA antibody production in the serum at 24 h after EP. The antibody concentration reached approximately 3,000 (= 10^3.53^) ng/ml after 120 h. On the other hand, by HD, we could first detect serum antibodies after 4 h. After 24 h, the antibody concentration reached approximately 20,000 (= 10^4.35^) ng/ml and this expression level was stable until after 120 h. Thus, HD can induce antibodies more rapidly and at a higher expression level than that by EP.

### HD Induces Neutralizing Antibodies in Mucosal Tissues

HD induced rapid and stable production of neutralizing antibodies in the serum. However, it was unknown whether HD could also induce anti-HA antibody production in the respiratory mucosa. Because IAV first attacks the host respiratory mucosa, production of neutralizing antibodies at this site is essential for a protective effect against influenza virus infection. We and other groups have previously reported that serum IgG antibodies were secreted in mucosal tissues and prevented virus infection (10, 33). To confirm these results, we conducted EP or HD in BALB/c mice. At 20 days (EP) (10) or 5 days (HD), which was the peak time point of anti-HA antibody production, serum, bronchoalveolar lavage without serum contamination, urine, and feces were obtained from the mice and anti-HA IgG levels were measured by quantitative ELISA. As shown in Figure 1B, both methods could induce anti-HA antibody expression in the respiratory mucosa. HD induced more than eightfold higher levels of serum antibodies (from 10^4.31^ to 10^5.24^) and approximately fivefold higher levels in the bronchoalveolar lavage (from 10^1.38^ to 10^2.07^) compared to those by EP. Interestingly, antibodies were detected even in the urine and fecal specimens from mice subjected to HD [approximately 20–30 (= 10^1.28^–10^1.41^) ng/ml] (Figure 1B). These results suggest that HD could induce high levels of neutralizing antibodies in several mucosal tissues.

### HD Reduces Influenza Virus Titer

**Figure 2:**
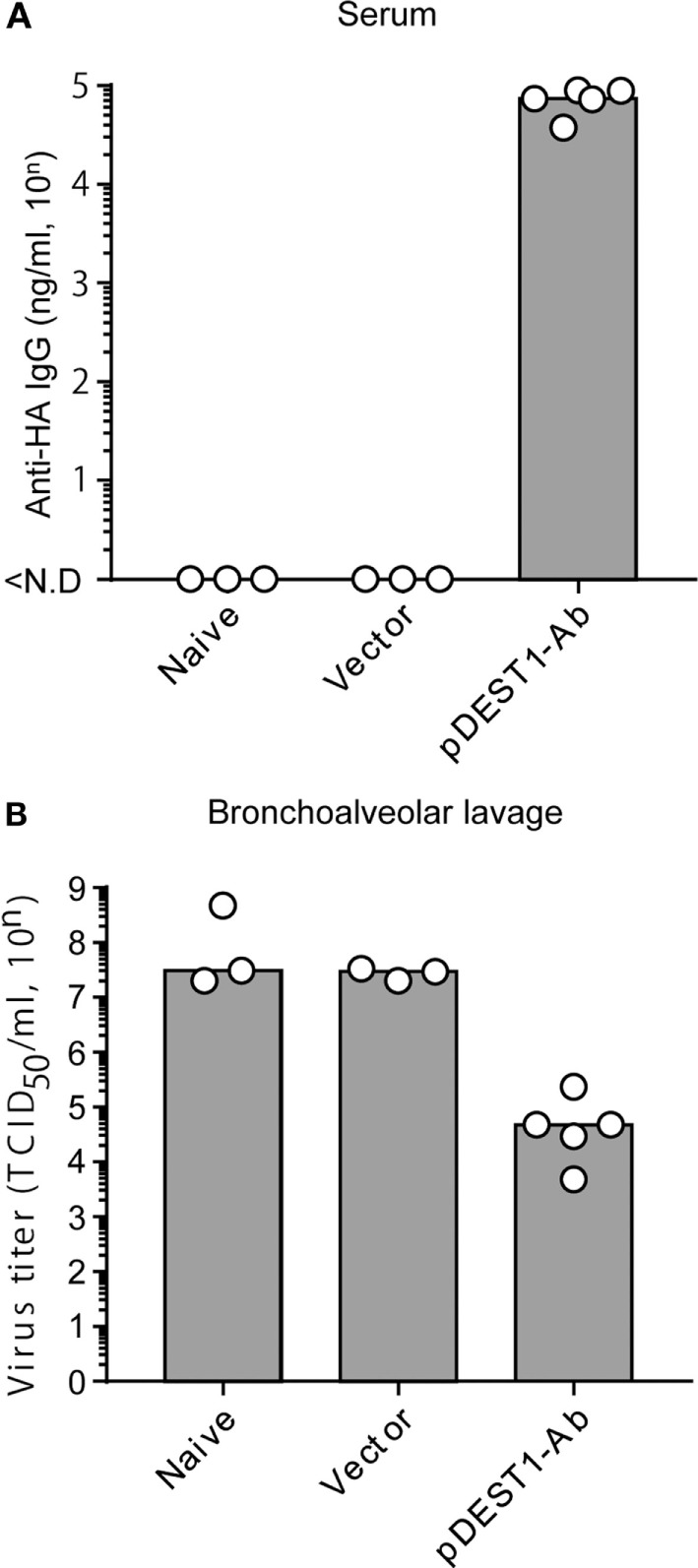
HD reduced virus titer in the bronchoalveolar lavage. **(A,B)** HD for anti-HA IgG was performed in BALB/c mice 1 day post-infection with a lethal dose of A/PR8 virus (1,000 TCID_50_). Three days post-infection, serum and bronchoalveolar lavage specimens were obtained from the infected mice. **(A)** Neutralizing monoclonal antibody (mAb) levels in the serum were measured by quantitative ELISA. ND, not detected. **(B)** Viral titers in the bronchoalveolar lavage specimens were determined by a micro-titer assay, as an index of protection against infection. Data were analyzed using a non-parametric Kruskal–Wallis test (*p* = 0.02204). Each bar represents the median. The indicated data are representative two independent experiments.

HD significantly induces rapid and potent neutralizing antibodies in the serum and in several mucosal tissues. To quantitatively assess the therapeutic efficacy of HD after IAV infection, virus titers were measured in bronchoalveolar lavage specimens. We first confirmed anti-HA antibody production in the serum after HD. As shown in Figure 2A, the serum anti-HA antibody levels reached approximately 70,000 ng/ml (= 10^4.86^ ng/ml). At this time, the viral titer in the bronchoalveolar lavage was reduced to approximately 1/400 (from 10^7.44^ to 10^4.86^) compared to that in the vector control group (Figure 2B). These data therefore indicate that a single HD administration induces neutralizing antibody production and that these antibodies control the proliferation of IAV.

### HD Confers Protection from Lethal IAV Infection

**Figure 3:**
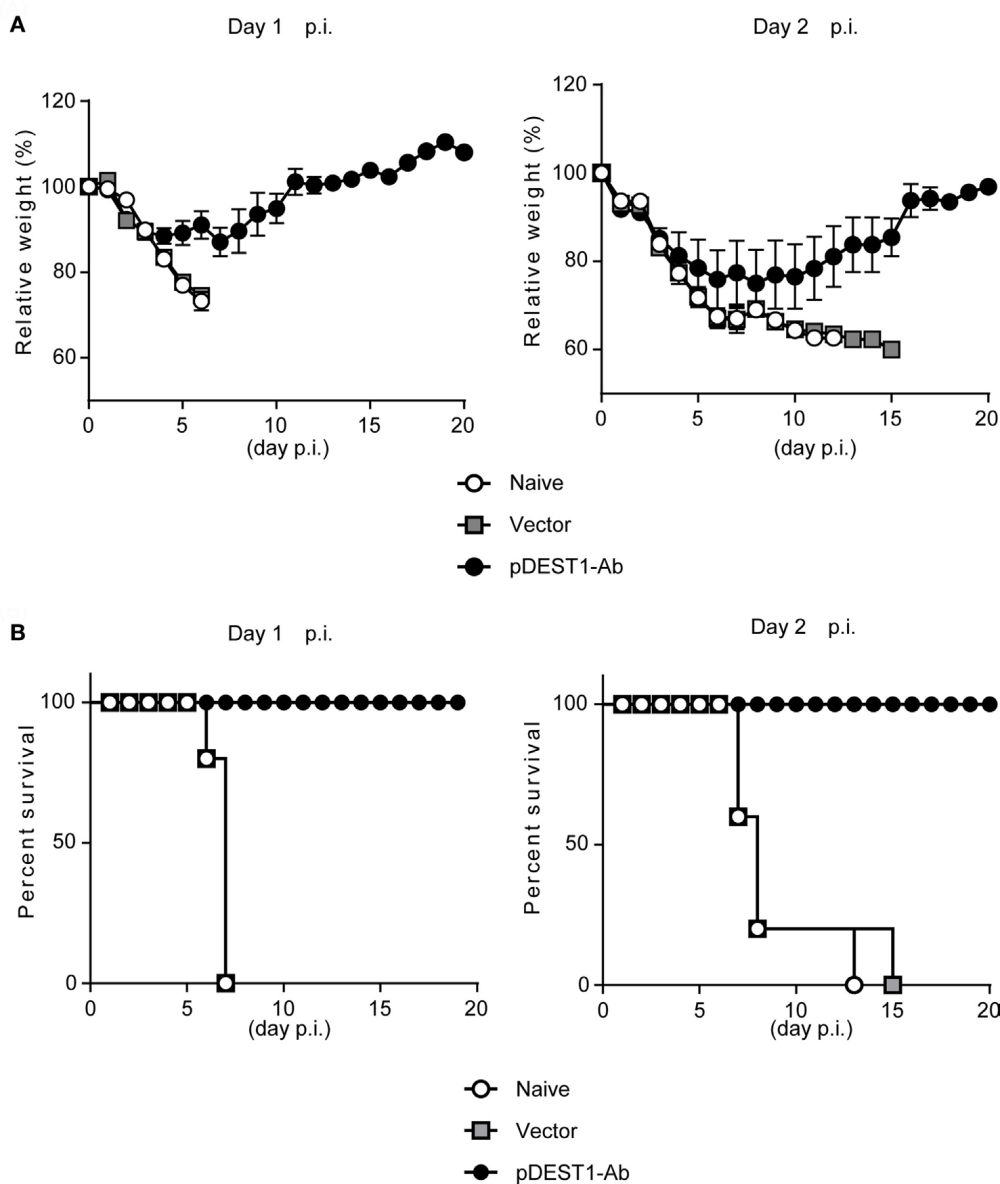
HD protects mice from a lethal dose of influenza virus infection. **(A,B)** BALB/c mice were subjected to HD with either pDEST1-empty (vector) or pDEST1-Ab encoding anti-HA IgG at 1 or 2 days post-infection with a lethal dose (1,000 TCID_50_) of A/PR8 virus (*n* = 5). The body weight **(A)** and survival rates **(B)** were monitored for 20 days. Body weight was expressed relative to the initial mean body weight of each group. Error bars represent the SEM. The indicated data from “Day 2 p.i.” are representative of two independent experiments.

To evaluate the therapeutic effect of HD after IAV infection, we infected BALB/c mice with a lethal dose of IAV. We then performed HD at one (Day 1 p.i.) or two (Day 2 p.i.) days post-infection. The changes in body weight (Figure 3A) and survival rates (Figure 3B) were monitored for 20 days. As shown in the left panels of Figures 3A,B (Day 1 p.i.), the body weight of all naive mice and empty vector transferred mice was decreased severely and all mice died within 7 days post-infection. In contrast, all mice subjected to HD survived even 20 days post-infection. These mice showed between 10 and 15% loss of body weight from 3 to 11 days post-infection, but by 12 days post-infection, all mice completely recovered from the weight loss. As shown in the right panels of Figure 3 (Day 2 p.i.), the body weight of all naive mice and empty vector transferred mice was decreased severely and all mice died within 15 days post-infection. In contrast, all mice subjected to HD survived even after 20 days post-infection. Some of these mice showed up to 25% loss of body weight from 1 to 19 days post-infection, but by 20 days post-infection, all mice had completely recovered from the weight loss. These results indicate that a single administration of HD can rescue mice from lethal IAV infection even at 2 days post-infection.

### Plasmid Encoding Anti-HA IgG, IgA, and IgM Neutralize IAV Infection *In Vitro*

**Figure 4:**
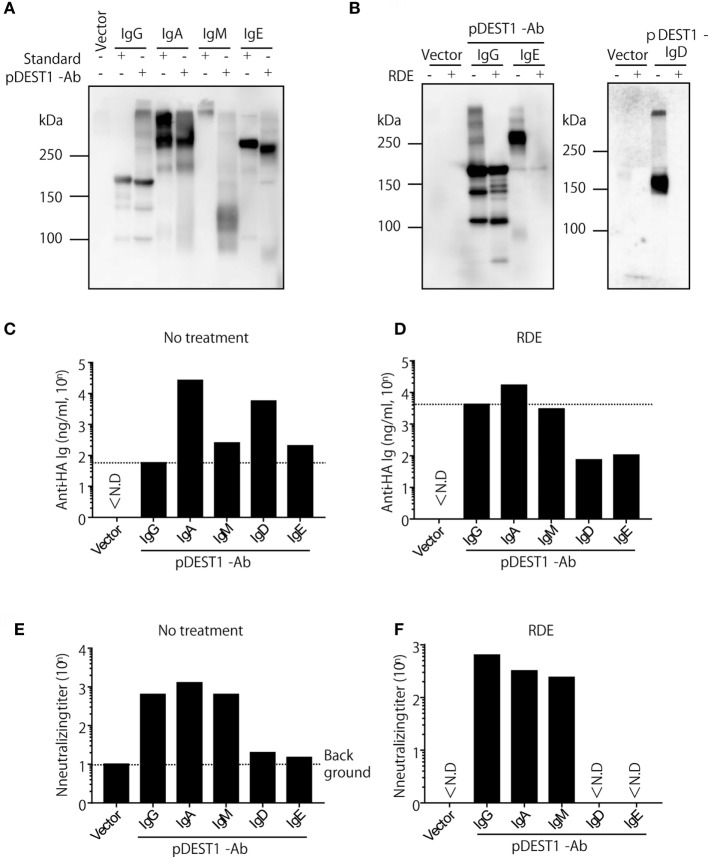
Anti-HA IgG, IgA, and IgM neutralize influenza virus *in vitro*. **(A–F)** HEK293T cells were co-transfected with pCADEST1-empty (vector) or pCADEST1-anti-HA kappa and anti-HA IgG, anti-HA IgA and Joining chain, anti-HA IgM and Joining chain, anti-HA IgD, or anti-HA IgE. Supernatants were collected after 1 week. **(B,D,F)** The supernatants were treated with receptor destroying enzyme (RDE) for overnight at 37°C. **(A,B)** The supernatants were processed by western blotting under non-reducing conditions followed by probing with HRP-conjugated goat anti-mouse IgG, IgA, IgM, and IgE. For detecting IgD, the blotted supernatants were probed with APC-conjugated rat anti-mouse IgD followed by HRP-conjugated anti-rat IgG. The indicated data are representative of two independent experiments. **(C,D)** The concentration of anti-HA antibodies in the supernatants was measured by competitive ELISA. A 96-well plate coated with HA was incubated with the supernatants, followed by addition of biotin-conjugated anti-HA IgG monoclonal antibody (mAb). **(E,F)** The neutralizing antibody titer was measured by a micro-neutralization assay. ND, not detected.

Our previous report and the current research indicate that anti-HA IgG induced by antibody gene delivery had a neutralizing effect against IAV infection (10, 34, 35). However, it is unknown whether other isotypes induced by antibody gene delivery can protect against IAV infection. To examine this, we first modified the plasmid encoding the anti-HA antibody. We genetically switched anti-HA IgG to anti-HA IgA, IgM, IgD, and IgE. In order to analyze the expression level of these isotypes *in vitro*, we transfected these plasmids into HEK293T cells and analyzed the antibody levels in the supernatants. As shown in Figure 4A, we observed specific bands for anti-HA IgG, anti-HA IgA, and anti-HA IgE. These bands were almost the same size as those of the respective standard samples. Although we could recognize a dimer of anti-HA IgA, polymeric IgM was not observed on this membrane (Figure 4A, lanes 4, 5, and 7), whereas the polymeric form was detected in standard IgM (Figure 4A, lane 6). On the other hand, in Figure 4B, we could recognize both monomeric and polymeric forms of IgD (Figure 4B, right panel, lane 3). We next quantified the antibody concentration in the supernatant by competitive ELISA. As shown in Figure 4C, production of anti-HA IgA, IgM, IgD, and IgE was found to be greater than that of IgG. The neutralizing titer of anti-HA IgA and IgM was almost equal to that of anti-HA IgG (Figure 4E). However, the neutralizing ability of anti-HA IgD and IgE was low and almost non-specific. Because influenza HA binds to sialic acid, RDE that is a sialidase from *Vibrio cholerae*, is usually added to avoid non-specific binding due to sialic acid present in the supernatant. Interestingly, the binding activity in anti-HA IgD and IgE was reduced with RDE (Figure 4D). Moreover, the neutralizing activity of anti-HA IgD and IgE was also completely reduced in the presence of RDE (Figure 4F). The reason for this inactivity was determined by western blot analysis. As shown in Figure 4B, anti-HA IgD (Figure 4B, right panel, lane 4) and IgE (Figure 4B, left panel, lane 6) were not detected upon treatment with sialidase. These data demonstrate that anti-HA IgG, IgA, and IgM possess neutralizing activity for IAV infection, whereas anti-HA IgD and IgE are easily inactivated by sialidase and consequently have no neutralizing activity.

### HD for Anti-HA IgG and IgA Induces Protection against Upper Respiratory Infection

**Figure 5:**
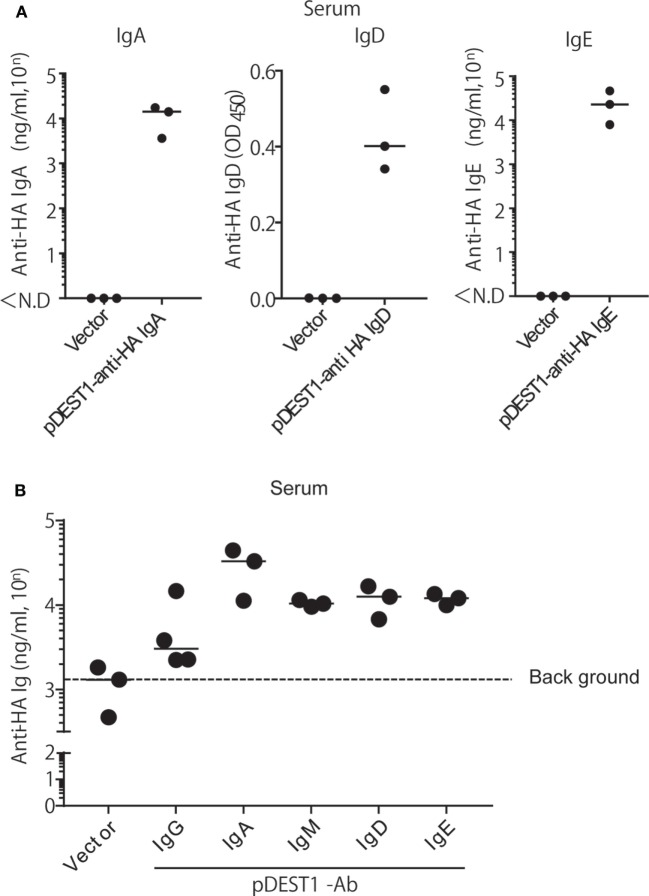
HD induces the expression of all isotypes of anti-HA antibodies in the serum. **(A)** HD was conducted in BALB/c mice and after 1 day, serum was obtained. The expression of each antibody isotype was determined by ELISA using HRP-conjugated got anti-mouse IgA, IgE, or APC-conjugated rat anti-mouse IgD followed by HRP-conjugated anti-rat IgG. **(B)** The expression level of each isotype was determined by competitive ELISA. A 96-well plate coated with A/PR8-HA, was incubated with the serum samples, followed by biotin-conjugated anti-HA IgG. Data were analyzed using a non-parametric Kruskal–Wallis test (*p* = 0.0457). Horizontal bar represents the median. The indicated data are representative of two independent experiments.

As mentioned above, we succeeded in expressing all classes of anti-HA Igs *in vitro*. Next, we evaluated whether all classes of anti-HA Igs are equally expressed *in vivo*. We performed HD for each class of anti-HA Ig in mice and collected the serum 1 day after injection. Other than IgM and the already demonstrated IgG (Figure 2A), anti-HA IgA, IgD, and IgE antibodies could be detected in the serum (Figure 5A). Anti-HA IgM antibody could also be evaluated but with a high level of background (Data not shown). To compare the expression levels of all classes of anti-HA antibodies, we performed competitive ELISA using the serum samples as shown in Figure 5B. The expression levels of anti-HA IgA, IgM, IgD, and IgE in the serum were equal at around 10^4^ ng/ml compared with that of anti-HA IgG [approximately 5,700 (= 10^3.76^) ng/ml] (Figure 5B). These data correlated with the results of the *in vitro* experiment (Figure 4C). We also evaluated the neutralizing antibody titers in the serum (Figure S2 in Supplementary Material). Experiments conducted using IgD and IgE were not effective in the *in vitro* neutralizing experiment (Figure 4E). Anti-HA IgG, IgA, and IgM in serum also indicated neutralizing potential.

In these experiments, we co-transfected the plasmid encoding the joining chain along with anti-HA IgA or anti-HA IgM. We then evaluated the importance of the joining chain for the expression of these antibodies. We transfected these plasmids with or without the joining chain expression vector into HEK293T cells and analyzed the antibody levels in the supernatants by western blotting. Each specific band was detected only under with the expression of joining chain (Figure S3A in Supplementary Material). This result suggests that the joining chain is important for anti-HA IgA and IgM expression. We also confirmed these results by performing HD with or without the joining chain in mice. As shown in Figure S3B in Supplementary Material, we could first detect serum antibodies both with and without the joining chain at 4 h after injection. After 24 h, the antibody concentration of IgG and IgA with the joining chain reached approximately 10^4^ ng/ml, but the level of IgA without joining chain could only be detected slightly, and was undetectable after 36 h (Figure S3B in Supplementary Material). These results suggest that the joining chain is important for the stable expression of anti-HA IgA.

**Figure 6:**
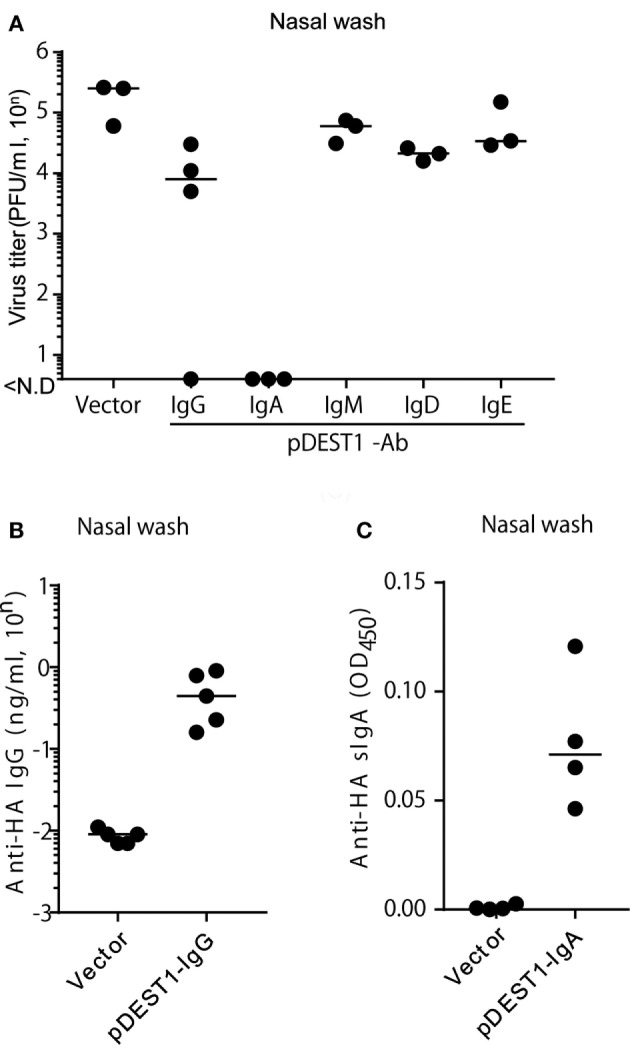
HD protects mice from influenza virus infection in the upper respiratory tract. **(A)** HD was conducted in BALB/c mice 8 h after intranasal infection (1,000 PFU/2 μl, each nostril). At 3 days post-infection, the virus titer in nasal wash specimens was determined by an MDCK plaque assay. Data were analyzed using a non-parametric Kruskal–Wallis test (*p* = 0.0091). **(B)** HD for anti-HA IgG was conducted in BALB/c mice and after 1 day, nasal wash specimens were obtained under serum-excluding conditions and analyzed for the concentration of anti-HA IgG by ELISA. **(C)** HD for anti-HA IgA was conducted in BALB/c mice after 8 h of virus infection. At 3 days post-infection, the nasal wash was obtained and the secreted HA-specific IgA (anti-HA sIgA) level was measured by ELISA using rabbit anti-mouse polymeric Ig receptor (pIgR). Horizontal bar represents the median. The indicated data are representative of two independent experiments.

Finally, we analyzed whether HD inhibits virus proliferation in the nasal cavity. We conducted HD in mice at 8 h post-infection and collected nasal wash specimens from these mice at 3 days post-infection. We then measured the virus titter in these specimens. As shown in Figure 6A, anti-HA IgG reduced the virus titer to 1/20 (from 10^5.27^ to 10^4.06^) compared with the vector control group. Interestingly, the virus titer was completely reduced with anti-HA IgA (Figure 6A). On the contrary, we could not recognize an inhibitory effect on the virus titer by the HD for anti-HA IgM, IgD, and IgE. To confirm protection in the nasal cavity, we analyzed the antibody concentration in nasal wash specimens. Anti-HA IgG (Figure 6B) and secreted HA-specific IgA (anti-HA sIgA) (Figure 6C) were detected in the nasal wash of several HD administered mice. We also confirmed that anti-HA IgA was detected in the serum only with the joining chain (Figure S4A in Supplementary Material) and induced almost complete reduction of virus titer in the nasal wash (Figure S4B in Supplementary Material). These data show that HD for anti-HA IgG and IgA could induce neutralizing antibodies in the nasal cavity and that these antibodies protected from IAV infection in the upper respiratory tract.

## Discussion

Here, we report that a single gene transfer encoding neutralizing antibodies by HD induces therapeutic effects even after a lethal dose of IAV infection. In our previous report, we suggested that the expression levels of antibodies correlated with the protective efficacy against IAV infection (10). We also showed that the virus titer in the bronchoalveolar lavage was reduced according to the concentration of the neutralizing antibody produced (10). In this study, we could clearly show that neutralizing antibody induction by HD was stronger than that by EP. We were able to show a curative effect in HD within 2 days after IAV infection because of rapid and potent antibody production.

We predict that the liver will be the main antibody-producing organ in HD. The liver is well-known as the organ synthesizing many serum proteins (36). Recent studies have shown that the liver is a major tissue of gene expression in the hydrodynamics procedure (11, 12). They also indicate that hydrodynamic injection transiently generates membrane pore in the hepatocyte, followed by gene delivery (37). These papers also support our prediction. Interestingly, HD offers the possibility of neutralizing antibody secretion in multiple mucosal tissues, as demonstrated by their presence in the bronchoalveolar lavage, nasal wash, urine, and feces. Other groups have also indicated that gene transfer by hydrodynamics could induce expression in the kidney, spleen, lung, and heart (11, 12). These data suggest that HD is also effective for viruses that infect other organs, such as enteroviruses.

Recently, Limberis et al. demonstrated intranasal antibody gene transfer using an adeno-associated virus (AAV) vector (34). The genetic delivery of therapeutic mAbs using AAV vectors for *in vivo* production offers a new potential therapy for microbial disease (34, 35, 38–41). This may eliminate the inconvenience of weekly mAb infusions over a long time. Although AAV has not been reported to cause harm in humans, there are many questions on whether artificial virus vectors are really safe because they could induce an immune response against the viral backbone (42–44). On the other hand, we succeeded in inducing neutralizing antibody production without using a viral vector. To our knowledge, this is the first report of neutralizing antibody secretion in the nasal mucosa by intravenous injection with a non-viral vector. HD is considered a safer alternative because it does not involve a viral vector. Moreover, the safety of plasmid transfer has been demonstrated in preclinical studies; potential integration into the host genome was shown to be negligible and was several orders of magnitude below the spontaneous mutation rate that occurs naturally in mammalian genes (45).

To particularly analyze which isotypes could reduce influenza virus infection in the upper respiratory tract, we established all classes of anti-HA antibodies and analyzed their virus neutralization effect (Figure 6A). Anti-HA IgG, IgA, IgM, IgD, and IgE induced from the plasmid showed almost the same expression level *in vitro* (Figure 4) and *in vivo* (Figure 5). To our knowledge, this study is the first report for the induction of all antibody isotypes in the animal body by gene transfer. Palladino et al. showed that passively transferred IAV-specific monoclonal IgM antibodies in SCID mice provide a prophylactic effect against IAV infection (46). However, we could not clearly demonstrate a therapeutic effect for upper respiratory infection by HD for anti-HA IgM (Figure 6A). From the western blot assay (Figure 4A), this was considered to be a result of the presence of monomeric IgM and absence of polymeric IgM in the supernatant or serum. Polymeric IgM can undergo transcytosis *via* the pIgR onto mucosal surfaces to provide protection from pathogenic invasion (47). Monomeric IgM might not undergo transcytosis *via* mucosal pIgR to reach the upper respiratory tract.

Although some researchers have shown that IAV-specific IgE is induced in human serum by the seasonal vaccine to possibly cause anaphylaxis (48, 49), its protective efficacy has not been evaluated. Our data indicate that it is difficult to protect against IAV infection using anti-HA IgD and IgE *in vitro* and *in vivo*. As shown in Figure 4, the binding activity of these antibodies was reduced by sialidase. Influenza virus possesses a sialidase known as neuraminidase. Previous reports have shown that IgD and IgE were deglycosylated by sialidase (50, 51). It is possible that anti-HA IgD and IgE were inactivated due to deglycosylation by sialidase. On the other hand, we demonstrated that HD induced around 10^4^ ng/ml of IgE in the serum. Using HD, a novel role of IgE antibodies can be demonstrated *in vivo*, for example, in protection against parasites and new therapies for allergies to block allergen-specific IgE antibodies from binding the Fcε receptor.

In this report, we demonstrate that HD for anti-HA IgG and IgA can also protect the upper respiratory tract from infection even after IAV exposure. We show that anti-HA IgG induced by HD are secreted in the upper respiratory tract. We have already shown the contribution of anti-HA IgG to the protective effect in the upper respiratory tract (10). Tamura et al. review that the IgG transude from the serum to the mucus by diffused mucosal cells and are largely distributed on the alveolar epithelia to prevent influenza virus infection (33). These data suggest that IgG passive immunotherapy can contribute to prevention of infection in both the upper and lower respiratory tracts. Furthermore, in the current study, we discovered that anti-HA IgA reduced the virus titer notably, to almost undetectable levels. In previous vaccine research, IgA is well-known as an important protective tool against IAV infection in the upper respiratory tract (33). Some researchers have also reported that IgA is important for protection against influenza virus (13–15), *Helicobacter felis* (52), or Sendai virus infection (53) by using passive IgA immunization. Leusen suggests that passive IgA immunization is effective in antibody-dependent cell-mediated cytotoxicity using whole blood mononuclear cells (54). We also indicated its effectiveness by a new method of passive immunization using gene-based mAbs. In the current study, we focused on IgA induction in the upper respiratory tract by HD. By the normal method of nasal wash collection, we cannot exclude contamination with serum IgA. Secretary IgA in the mucus lumen usually binds to the secretory component (SC) whose precursor is pIgR, *via* the joining chain (55). Asahi et al. proposed IAV-specific IgA production in the nasal wash using a polyclonal antibody against a SC (31). As shown in Figure 6C, we similarly demonstrated the secretion of HA-specific IgA (anti-HA sIgA) in the nasal wash. These data indicate that we could obtain secretory neutralizing IgA in the nasal mucosal tissue by HD. The joining chain was revealed to be important for the expression of anti-HA IgA and protection against IAV infection (Figures S3 and S4 in Supplementary Material).

In preclinical trials of gene transfer by hydrodynamic injection, liver-targeted hydrodynamic gene delivery in dogs using a computer-controlled device has been reported (56). The report indicated that no systemic damage or toxicity was observed. Physiological parameters also remained in the normal ranges during and after hydrodynamic injection. Other groups presented hydrodynamic injection in the larger animal model of the pig (57–60), and rhesus macaque (61). Hydrodynamic injection will be less effective in pigs than in rodents, possibly because of larger liver size or a less compliant connective tissue framework (58). However, Fabre et al. showed that the inferior vena cava segment approach is a clinically acceptable approach to pig liver gene therapy (58). Although the gene delivery by hydrodynamic injection succeeded in clinical trial, it seems difficult to keep the medical benefit (57). Khorsandi et al. injected 1–45 mg plasmid DNA of human thrombopoietin in volumes up to 300 ml into cirrhotic patient. All patients tolerated injection without any obvious adverse effect. However, a clinically useful elevation of platelet count was not found (51). As Zhang et al. and AI-Dosari et al. described (37, 62), it is important to establish new medical devices (e.g., specific catheters and occlusion balloons) under the principles of hydrodynamics injection. For clinical safety, local gene delivery is also needed to avoid exacerbating diseases symptoms. From these researches, HD modified as clinical application will be useful for the prevention of complication and aggravation such as pneumonia or encephalitis by virus infections. Moreover, this technique is a possible therapeutic strategy of antibody-drugs for cancer and autoimmune disease.

In summary, HD provides rapid and potent neutralizing antibodies for a therapeutic effect after both lethal IAV infection and upper respiratory infection. Although several reports demonstrate that neutralizing antibodies induced by gene transfer provide a prophylactic effect against pathogens (63), in this paper, we have established passive immunotherapy along with gene therapy after virus infection for the first time. We also demonstrated that HD could induce antibody secretion in the serum as well as in multiple mucosal organs. HD modified for clinical application can be used for passive immunotherapy to induce antibodies immediately in case of pandemic emergencies.

## Ethics Statement

This study was carried out in accordance with the recommendations of “the animal facility at Tokyo University of Science and Aichi Medical University.” The protocol was approved by the “animal facility at Tokyo University of Science and Aichi Medical University.”

## Author Contributions

TY contributed to the study design, performed most experiments, analyzed and interpreted data, and drafted the manuscript. HH and AA provided tools and reagents. MN contributed to the study design. DN, AF, II, HT, and NM assisted with experiments and the study design. KM analyzed the experimental statistics. JC conceived and supervised this project. SA-T contributed to the study design and supervised the manuscript. All authors reviewed and contributed to the manuscript.

## Conflict of Interest Statement

The authors declare that the research was conducted in the absence of any commercial or financial relationships that could be construed as a potential conflict of interest.
