# Supplementary material for: Neutralizing Antibodies Induced by Gene-Based Hydrodynamic Injection Have a Therapeutic Effect in Lethal Influenza Infection
Source: Front Immunol. 2018 Jan 24;9:47. doi: 10.3389/fimmu.2018.00047 (PMC5787536; doi:10.3389/fimmu.2018.00047)
Supplement: Supplementary file 1 [file Data_Sheet_1.PDF]

frontiers

Supplementary Data

Neutralizing antibodies induced by gene-based hydrodynamic injection have a therapeutic effect in lethal Influenza infection

Tatsuya Yamazaki[1,2], Maria Nagashima[2], Daisuke Ninomiya[2], Akira Ainai[3], Akira Fujimoto[2], Isao Ichimonji[1], Hidekazu Takagi[1], Naoko Morita[1], Kenta Murotani[4], Hideki Hasegawa[3], Joe Chiba[2], and Sachiko Akashi-Takamura[1*]

[1]Department of Microbiology and Immunology, Aichi Medical University, School of Medicine, 1-1 Yazakokarimata, Nagakute, Aichi, Japan
[2]Department of Biological Science and Technology, Tokyo University of Science, Niijuku 6-3-1, Katsushika-ku, Tokyo Japan
[3]Department of Pathology, National Institute of Infectious Diseases, Shinjuku-ku, Tokyo 162-8640, Japan
[4]Division of Biostatistics, Clinical Research Center, Aichi Medical University, School of Medicine, 1-1 Yazakokarimata, Nagakute, Aichi, Japan

*** Correspondence:** Sachiko Akashi-Takamura; E-mail: sachiko@aichi-med-u.ac.jp

Supplementary Data|

We indicate amino acid sequences of the mouse anti-HA heavy chain (IgG, IgA, IgM, IgD, IgE), kappa chain, and joining chain. The signal sequence and variable region were determined by SOSUI (http://harrier.nagahama-i-bio.ac.jp/sosui/) and ImMunoGeneTics (IMGT, http://www.imgt.org/).

Amino acid sequence of mouse anti-HA IgG

MKCSWIMFFLMAVVTGVNAEVQLQQSGAEFVKPGASVKLSCTGSGFNIKDTYMHWVKQRP 60

EQGLVRIGRIDPANGDTKYDPKFQGKATITADTSSNTAYLQLSSLTSEDTAVYYCARSHF 120

YKLRYWYFEVWGAGTAVTVSSAKTTPPSVYPLAPGSAAQTNSMVTLGCLVKGYFPEPVTV 180

TWNSGSLSSGVHTFPAVLQSDLYTLSSSVTVPSSTWPSETVTCNVAHPASSTKVDKKIVP 240

RDCGCKPCICTVPEVSSVFIFPPKPKDVLTITLTPKVTCVVVDISKDDPEVQFSWFVDDV 300

EVHTAQTQPREEQFNSTFRSVSELPIMHQDWLNGKEFKCRVNSAAFPAPIEKTISKTKGR 360

PKAPQVYTIPPPKEQMAKDKVSLTCMITDFFPEDITVEWQWNGQPAENYKNTQPIMDTDG 420

SYFVYSKLNVQKSNWEAGNTFTCSVLHEGLHNHHTEKSLSHSPGK*

Dotted line: Signal sequence

Double line: Variable region

Bold line: Constant region

*: Stop codon

Amino acid sequence of mouse anti-HA IgA

MKCSWIMFFLMAVVTGVNAEVQLQQSGAEFVKPGASVKLSCTGSGFNIKDTYMHWVKQRP 60

EQGLVRIGRIDPANGDTKYDPKFQGKATITADTSSNTAYLQLSSLTSEDTAVYYCARSHF 120

YKLRYWYFEVWGAGTAVTVSSESARNPTIYPLTLPPALSSDPVIIGCLIHDYFPSGTMNV 180

TWGKSGKDITTVNFPPALASGGRYTMSSQLTLPAVECPEGESVKCSVQHDSNPVQELDVN 240

CSGPTPPPPITIPSCQPSLSLQRPALEDLLLGSDASITCTLNGLRNPEGAVFTWEPSTGK 300

DAVQKKAVQNSCGCYSVSSVLPGCAERWNSGASFKCTVTHPESGTLTGTIAKVTVNTFPP 360

QVHLLPPPSEELALNELLSLTCLVRAFNPKEVLVRWLHGNEELSPESYLVFEPLKEPGEG 420

ATTYLVTSVLRVSAETWKQGDQYSCMVGHEALPMNFTQKTIDRLSGKPTNVSVSVIMSEG 480

DGICY*

Dotted line: Signal sequence

Double line: Variable region

Bold line: Constant region

*: Stop codon

Amino acid sequence of mouse anti-HA IgM

MKCSWIIFFLMAVVTGVNAEVQLQQSGAEFVKPGASVKLSCTGSGFNIKDTYMHWVKQRP 60

EQGLVRIGRIDPANGDTKYDPKFQGKATITADTSSNTAYLQLSSLTSEDTAVYYCARSHF 120

YKLRYWYFEVWGAGTAVTVSSSQSFPNVFPLVSCESPLSDKNLVAMGCLARDFLPSTISF 180

TWNYQNNTEVIQGIRTFPTLRTGGKYLATSQVLLSPKSILEGSDEYLVCKIHYGGKNRDL 240

HVPIPAVAEMNPNVNVFVPPRDGFSGPAPRKSKLICEATNFAPKPITVSWLKDGKLVESG 300

FTTDPVTIENKGSTPQTYKVISTLTISEIDWLNLNVYTCRVDHRGLTFLKNVSSTCAASP 360

STDILTFTIPPSFADIFLSKSADLTCLVSNLATYETLNISWASQSGEPLETKIKIMESHP 420

NGTFSAKGVASVCVEDWNNRKEFVCTVTHRDLPSPQKKFISKPNEVHKHPPAVYLLPPAR 480

EQLNLRESATVTCLVKGFSPADISVQWLQRGQLLPQEKYVTSAPMPEPGAPGFYFTHSIL 540

TVTEEEWNSGETYTCVVGHEALPHLVTERTVDKSTGKPTLYNVSLIMSDTGGTCY*

Dotted line: Signal sequence

Double line: Variable region

Bold line: Constant region

*: Stop codon

Amino acid sequence of mouse anti-HA IgD

MKCSWIIFFLMAVVTGVNAEVQLQQSGAEFVKPGASVKLSCTGSGFNIKDTYMHWVKQRP 60

EQGLVRIGRIDPANGDTKYDPKFQGKATITADTSSNTAYLQLSSLTSEDTAVYYCARSHF 120

YKLRYWYFEVWGAGTAVTVSSDKKEPDMFLLSECKAPEENEKINLGCLVIGSQPLKISWE 240

PKKSSIVEHVFPSEMRNGNYTMVLQVTVLASELNLNHTCTINKPKRKEKPFKFPESWDSQ 180

SSKRVTPTLQAKNHSTEATKAITTKKDIEGAMAPSNLTVNILTTSTHPEMSSWLLCEVSG 240

FFPENIHLMWLSVHSKMKSTNFVTANPTPQPGGTFQTWSVLRLPVALSSSLDTYTCVVEH 360

EASKTKLNASKSLAISGCYHLLPESDGPSRRPDGPALA*

Dotted line: Signal sequence

Double line: Variable region

Bold line: Constant region

*: Stop codon

Amino acid sequence of mouse anti-HA IgE

MKCSWIIFFLMAVVTGVNAEVQLQQSGAEFVKPGASVKLSCTGSGFNIKDTYMHWVKQRP 60

EQGLVRIGRIDPANGDTKYDPKFQGKATITADTSSNTAYLQLSSLTSEDTAVYYCARSHF 120

YKLRYWYFEVWGAGTAVTVSSSIRNPQLYPLKPCKGTASMTLGCLVKDYFPGPVTVTWYS 180

DSLNMSTVNFPALGSELKVTTSQVTSWGKSAKNFTCHVTHPPSFNESRTILVRPVNITEP 240

TLELLHSSCDPNAFHSTIQLYCFIYGHILNDVSVSWLMDDREITDTLAQTVLIKEEGKLA 300

STCSKLNITEQQWMSESTFTCKVTSQGVDYLAHTRRCPDHEPRGVITYLIPPSPLDLYQN 360

GAPKLTCLVVDLESEKNVNVTWNQEKKTPVSASQWYTKHHHNATTSITSILPVVAKDWIE 420

GYGYQCIVDHPDFPKPIVRSITKTPGQRSAPEVYVFPPPEEESEDKRTLTCLIQNFFPED 480

ISVQWLEDGKLISNSHHSTTTPLKSNGSNQGFFIFSRLEVAKTLWTQRKQFTCQVIHEAL 540

QKPRKLEKTISTSLGNTSLRPS*

Dotted line: Signal sequence

Double line: Variable region

Bold line: Constant region

*: Stop codon

Amino acid sequence of mouse anti-HA kappa

MDFQVQIFSFLLMSASVIMSRGQIVLTQSPALMSASPGEKVTMTCSASSSVSYMYWYQQK 60

PRSSPKPWILLTSNLASGVPVRFSGSGSGTSYSLTISSMEAEDAATYYCQQWSSNPPTFG 120

GGTKLEIRRADAAPTVSIFPPSSEQLTSGGASVVCFLNNFYPKDINVKWKIDGSERQNGV 180

LNSWTDQDSKDSTYSMSSTLTLTKDEYERHNSYTCEATHKTSTSPIVKSFNRNEC*

Double line: Variable region

Bold line: Constant region

*: Stop codon

Amino acid sequence of mouse joining chain

MKTHLLLWGVLAIFVKAVLVTGDDEATILADNKCMCTRVTSRIIPSTEDPNEDIVERNIR 60

IVVPLNNRENISDPTSPLRRNFVYHLSDVCKKCDPVEVELEDQVVTATQSNICNEDDGVP 120

ETCYMYDRNKCYTTMVPLRYHGETKMVQAALTPDSCYPD*

Dotted line: Signal sequence

*: Stop codon
