# Supplementary material for: Neutralizing Antibodies Induced by Gene-Based Hydrodynamic Injection Have a Therapeutic Effect in Lethal Influenza Infection
Source: Front Immunol. 2018 Jan 24;9:47. doi: 10.3389/fimmu.2018.00047 (PMC5787536; doi:10.3389/fimmu.2018.00047)
Supplement: Supplementary file 2 [file Data_Sheet_2.PDF]

Neutralizing antibodies induced by gene-based hydrodynamic injection have a therapeutic effect in lethal Influenza infection

Tatsuya Yamazaki[1,2], Maria Nagashima[2], Daisuke Ninomiya[2], Akira Ainai[3], Akira Fujimoto[2], Isao Ichimonji[1], Hidekazu Takagi[1], Naoko Morita[1], Kenta Murotani[4], Hideki Hasegawa[3], Joe Chiba[2], and Sachiko Akashi-Takamura[1*]

[1]Department of Microbiology and Immunology, Aichi Medical University, School of Medicine, 1-1 Yazakokarimata, Nagakute, Aichi, Japan
[2]Department of Biological Science and Technology, Tokyo University of Science, Niijuku 6-3-1, Katsushika-ku, Tokyo Japan
[3]Department of Pathology, National Institute of Infectious Diseases, Shinjuku-ku, Tokyo 162-8640, Japan
[4]Division of Biostatistics, Clinical Research Center, Aichi Medical University, School of Medicine, 1-1 Yazakokarimata, Nagakute, Aichi, Japan

*** Correspondence:** Sachiko Akashi-Takamura; E-mail: sachiko@aichi-med-u.ac.jp

Supplementary Figure 1

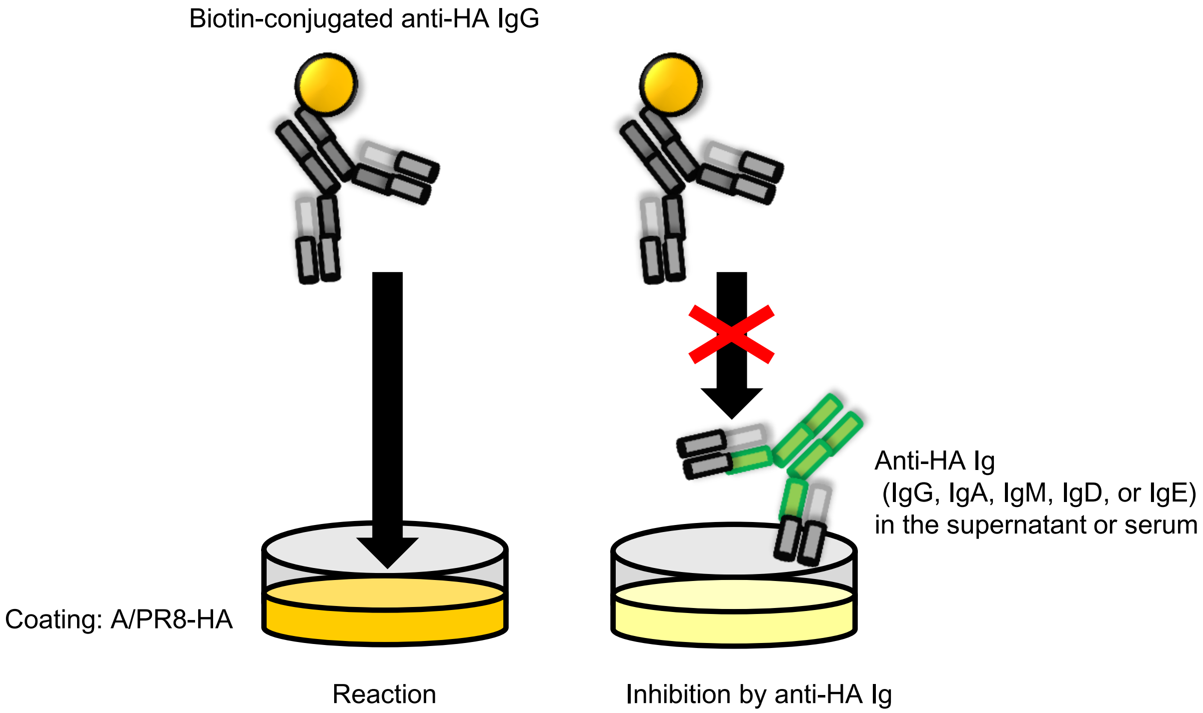

Supplementary Figure 1| Method of competitive ELISA

A 96 well plate coated with purified A/PR8-HA were incubated with the supernatant or serum, followed by incubation with biotin conjugated anti-HA IgG mAb. The titer was determined by inhibition curve based on absorbance of standard anti-HA IgG antibodies.

Supplementary Figure 2

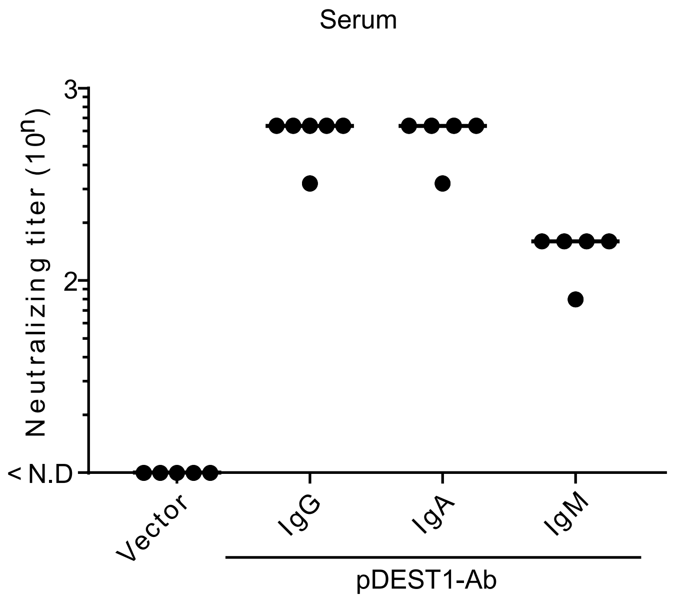

Supplementary Figure 2| ***In vitro*** **neutralizing titer of the serum**
HD was conducted in BALB/c mice as indicated. After 1 day, the serum was obtained and analyzed neutralizing titer by neutralizing assay as indicated in materials and methods. The titer was defined as the highest dilution that indicated no CPE. Data was analyzed by non-parametric Kruskal-Wallis test ($p=0.0004$). Horizontal bar represents the median.

Supplementary Figure 3

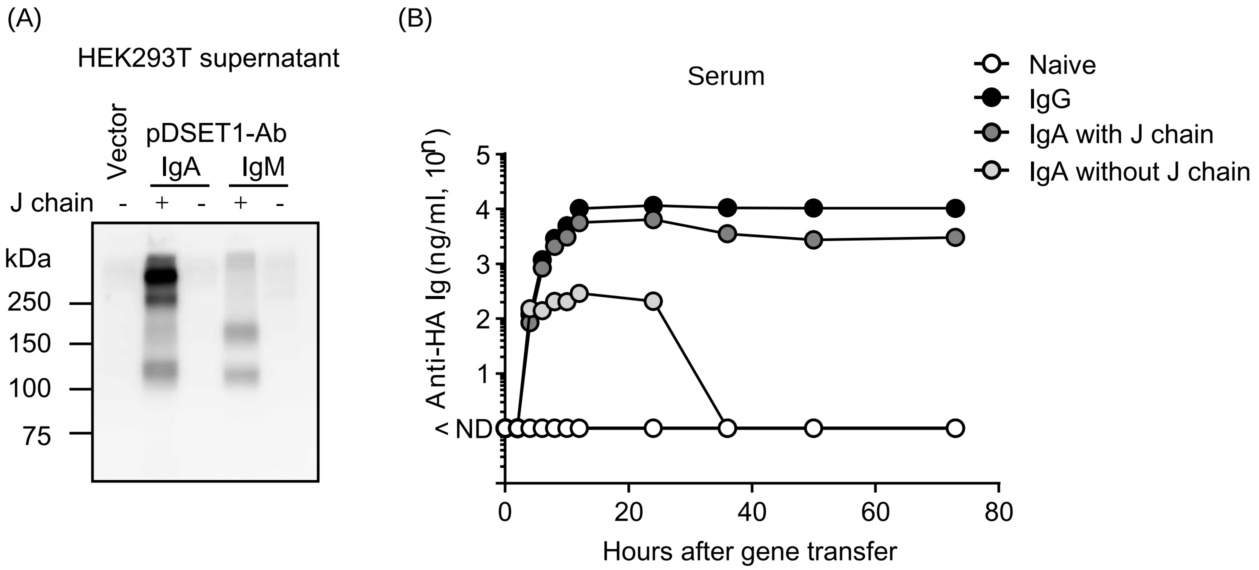

Supplementary Figure 3| Joining chain was important for the expression of IgA
(A) HEK293T cells were transfected with pCADEST1-empty (Vector), anti-HA IgA, and anti-HA IgM with pDEST1-Joining chain or not as indicated. One week later, the supernatants were obtained, and were processed by western blotting under non-reducing conditions, followed by immunoprobing with HRP-conjugated goat mouse anti- IgA and IgM. (B) Balb/c mice were injected with plasmid vectors encoding anti-HA IgA with Joining chain or not. Serum specimens were obtained at indicated times after administration. The serum titer was determined by quantitative ELISA using goat anti-mouse light chain kappa. Error bars represent the S.E.M. (n = 5).

Supplementary Figure 4

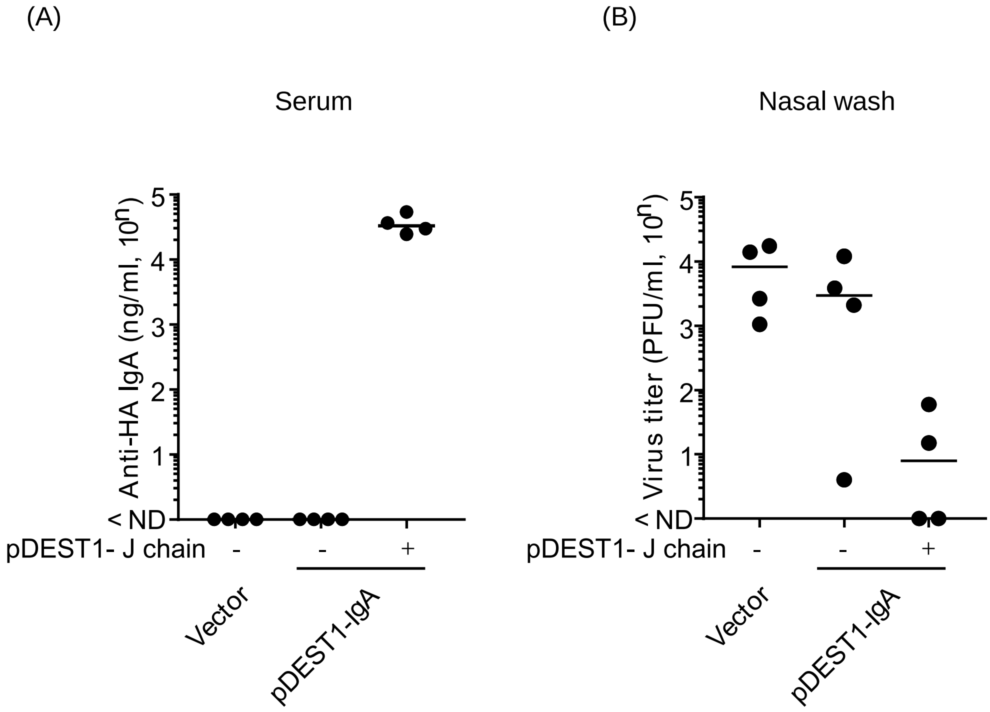

Supplementary Figure 4| The expression of anti-HA IgA and joining chain was important for the protection against IAV infection

HD of anti-HA IgA with or without joining chain was conducted in BALB/c mice 8 hours after intranasal infection (1000 PFU/2 µl, each nostril). At 3 days post-infection, the serum (A) and nasal wash (B) were obtained. (A) Titer of anti-HA IgA was determined by quantitative ELISA. (B) The virus titer in nasal wash specimens was determined by the MDCK plaque assay. Data was analyzed by non-parametric Kruskal-Wallis test (A: $p=0.0061$, B: $p=0.0317$). Horizontal bar represents the median.

Supplementary Table 1 |
The following primers were used for cloning indicated gene.

Detail	Sequence
Joining chain Forward	5'-CACCATGAAGACCCACCTGCTTCTCT-3'
Joining chain Reverse	5'-CTAGTCAGGGTAGCAAGAAT-3'
IgA for anti-HA Forward	5'-CGCAGGGACCGCGGTCACCGTCTCCTCAGAGTCTGCGAGAAATCCCACC-3'
IgA for anti-HA Reverse	5'-CTAGTAGCAGATGCCATCTCCCTCTGA-3'
IgM for anti-HA Forward	5'-GGACCGCGGTCACCGTCTCCTCAAGTCAGTCCTTCCCAAATGT-3'
IgM for anti-HA Reverse	5'-AGGCGCGCCTCAATAGCAGGTGCCGCCTGTGT-3'
IgD for anti-HA Forward	5'-GGACCGCGGTCACCGTCTCCTCAGATAAAAAGGAACCTGACAT-3'
IgD for anti-HA Reverse	5'-AGGCGCGCCTCAAGCTTTATGACTAGTCCTGG-3'
IgE for anti-HA Forward	5'-GGACCGCGGTCACCGTCTCCTCATCTATCAGGAACCCTCAGCT-3'
IgE for anti-HA Reverse	5'-AGGCGCGCCTCAGGAGGGACGGAGGGAGGTGT-3'
